# Research on the extraction of total saponins from hazel mushrooms via an ultrasound-assisted extraction method and their antitumor activity

**DOI:** 10.3389/fnut.2026.1809982

**Published:** 2026-03-25

**Authors:** Yuan Fang, Yilizhati Yimamu, Xinyu Cui, Wenjun Kan, Yanan Luo, Hao Wu

**Affiliations:** School of Laboratory Medicine (Pharmaceutical Sciences), Jilin Medical University, Jilin City, Jilin, China

**Keywords:** antitumor activity, hazel mushroom, medicinal food, saponins, UAE

## Abstract

Currently, “medicinal food” is a trending topic of discussion given the various bioactive compounds found within edible mushrooms. However, research on the collection and biological potency of saponins extracted from hazel mushroom, a species of *Armillaria*, is lacking. We aimed to conduct additional studies with respect to our previous endeavor, which was an effort to determine possible methods that can be used to alleviate ultrasonic-assisted extraction conditions of compounds and determine possible anticancer effects. Response surface methodology can be used to identify good extraction parameters, such as the ethanol concentration 73.28%, solid/liquid ratio 1:24.88 g/mL, and time 12.5 min, to obtain a maximum value of 21.4%. After extraction, we analyzed specific patterns of some saponins via ultrahigh performance liquid chromatography/ quadrupole time-of-flight mass spectrometry, which meant that these compound act on tumor cells. This study was conducted to determine the effect of such compounds on cancer.

## 1 Introduction

The Changbai mountain temperate forest ecosystem lies 41° north to 42° north. Its peculiar updown atmosphere and extremely fertile dark soil are referred to as chernozem, which allows this ecosystem to accommodate various large hazel mushrooms, similar to *Armillaria* spp. family Physalaciaceae, order Agaricales. Hazel mushrooms have been considered a necessity and is frequently linked with the three treasures of Northeast China. Biochemical analysis has verified its two natures: one as a food and the other as medicine. These fungi accumulate protein and essential amino acids, polysaccharides, etc., with specific bioactive compounds, such as saponins and armillarisin, having nutritional and therapeutic properties. According to previous studies, various types of *Armiralla* offer several biological benefits, such as antioxidation and tumor effects, and strengthen the immune system. The use of the neurological system as sedatives and anticonvulsants shows promise. However, its consumption by local folks has only begun. Old drugs can be removed by a reliable technology that can handle the source. Consequently, useful products can be constantly developed. Here, we confirmed the value of hazel mushrooms from Changbai mountain temperate forest ecosystem, providing an anchor point for the manufacture of a genuinely healthy product.

Edible mushrooms are part of an old still-current concept indicating that similar to medicine and food, drugs and foods can collaboratively serve one role, that is, function foods. These products gained attention when consumed in substantial amounts to gain true influence on healing. In addition to serving as food, these mushroom store interesting bioproducts, such as polysaccharides, proteins, sterols, polyphenols, and saponins (1–4). The developments in the fields of biology and chemistry, with respect to natural products, are focused on these small yet very crucial ingredients. Such an approach allows the maximum usage of forest landscape for greater industries, which currently include health. Saponins consist of sapogenins with sugar units, and they occur everywhere across plants and certain types of mushrooms. Available antitumor therapies especially control the immunity against tumors and address oxidative stress and metabolism (5, 6). Studies used mostly plant-based sources, such as ginseng (7–9) and liquor; however, a few have investigated fungus-based saponins. Structure of fungus-derived saponins provide fresh perspectives on nature–product chemistry. Compared with the plant-derived types, the saponin of most edible mushrooms is more common and unique. The release of compounds presents difficulty, mostly because of the complexity of the mycelium wall. The extraction of fungal saponins presents a challenge and is avoided by pharmaceutical and food businesses. For instance, specific triterpenoid saponins are the primary components accountable for the antiphlogistic and liver-protective effect observed on certain fungi (10, 11). Currently, with the use of edible fungal saponins, problems concerning yields and a lack of optimization in the extraction processes arise. In addition, solvent reflow is limited by the large volume used and long duration, which may harm temperature-sensitive substances. Scientists are exploring greener and better technology, such as ultrasound assistance or enzyme drawing, and meticulously observe the cleanliness and biological activity of extracts to gain understanding of the medicinal qualities of edibles and to create value.

With respect to the problems associated with older methods, ultrasonic-assisted extraction (UAE) is becoming an important green technology for dealing with natural phenomena (12, 13). In this method, ultrasonicated cavitation breaks into microjets, which allow the fungal cell wall to be opened more easily by the solvent. It can also accelerate the process to regain the desired material quickly and lower heat usage, which means that fine substances that are not shattered too rapidly by extreme heat (14), such as saponins, are protected. Polysaccharide and polyphenol studies revealed the ability of wild *Armillaria* to grow around Mt. Changbai. However, its saponin component remains unknown. Ultrasonics work efficiently in terms of mass transfer. The amount of power used and the time of application and the proportion of liquids and solids or the alcohol volume percentage can be adjusted. However, these optimal circumstances are not meant only for increasing efficacy; rather, they offer a feasible outline upon which to scale up this production system, supplying a starting position toward all works pertaining to structure–activity associations and food functionality. However, the RSM is not a single idea. Similar to Box–Behnken design (BBD), CCD allow for a deeper understanding of all sorts and parts. RSM gives more information on other interactions. The experimental raw data fitting to a quadratic regression equation were used to create a three-dimensional (3D) surface plot and contours showing how yields varied based on conditions (15, 16). The saponin from *Armillaria* displays complicated nonlinear interactions; hence, RSM can be used to find a good mix for maximum output. To make them both efficient and practical, this paper determined the exact value of a very suitable polynomial model.

There is a clear need to integrate ultrasound-assisted extraction (UAE) with response surface methodology (RSM) (17, 18) to optimize the recovery of saponins from hazel mushrooms. This combined approach could significantly enhance extraction efficiency and yield, offering a more robust alternative to existing methods. By using high-resolution UPLC/Q-TOF-MS, we aimed to determine what came from our work and the possibilities for killing cancer cells.

## 2 Materials and methods

### 2.1 Materials

Hazel mushroom (*Armillaria mellea*) samples were obtained from Changbai Mountain, Jilin Province, China. For preparation, we dried the samples until they reached a certain weight, ground them finely, and ran them through a 50-mesh screen to achieve a consistent texture. The powder was stored at 4 °C for the next step.

Human lung cancer (A549) and osteoma (MG63) cell lines were obtained from stocks within the lab. All the experimental solutions were prepared with ultrapure water. All the reagents and media involved in these experiments were of high-purity grade. The Medical Ethics Committee of Jilin Medical University has determined that the experimental protocols and objectives involving human/animal participants in this study adhere to ethical standards and international conventions. The A549 human lung cancer cell line and the MG63 osteoma cell line utilized in this study were procured from Meilun Biotechnology Co., Ltd. (Dalian, China).

### 2.2 Preparation of sample solutions of hazel mushroom extract via the UAE method

The extraction started with the addition of 2.0 g dried hazel mushroom powder to a 100 mL three-necked flask. Saponins were extracted via UAE, the ethanol concentration was adjusted from 50 to 90%, and the L/S ratio was changed from 1:10–1:30 g/mL. Then, the mixture was filtered and concentrated via rotary evaporation until dry. For protein removal, the residue was treated with a mixture of 100 mL absolute ethanol and 20 mL distilled water and then allowed to precipitate for 12 h. Finally, the solution was vacuum-filtered and evaporated again. The residue was taken up in 20 mL distilled water and transferred into a 250-mL separatory funnel with the same quantity of n-butanol saturated with water. The reaction mixture was shaken well and allowed to sit for 12 h to ensure separation of all the phases. The top n-butanol layer was then collected and dried by evaporation. Finally, the isolated saponins were redissolved in methanol and adjusted to a fixed volume to prepare the sample for analysis.

### 2.3 Determination of total saponin content (perchloric acid colorimetric method)

The perchloric acid colorimetric method was used to create quantification standards, with saponin aglycone as reference. After evaporation, 1.0 mL of methanol was introduced, and the reaction mixture was allowed to dry. A total of 5.0 mL perchloric acid was added at 70 °C for 15 min to ensure that all colors would appear. The absorbances were measured at 327 nm after cooling down the sample. Through the construction of the standard curve via a linear regression equation on the collected data, effective support can be given toward concentrating on the determination process when considering hazel mushroom saponin samples with particular mass fractions (Equation 1):

$$Y=\frac{B\times 20\times 20}{m\times 1000}\times 100\%$$ (1)

where Y: saponin extraction rate (%), B: concentration of the sample (mg/mL), and m: hazel mushroom quality (g).

### 2.4 Box–Behnken design (BBD) optimization

During our experimentation, only this change was applied to determine whether more hazel mushroom saponins can be obtained using ultrasound. The changes in the amount of alcohol present (X_1_: 50–90%), the time consumed for extraction (X_2_: 5–25 min), and the proportion of solids to liquids (X_3_: 1:10–1:30) were determined. This method involved keeping other parameters unchanged but changing the target and taking the saponin extraction rate as the standard to determine the parameter most suitable among various process parameter settings.

Considering one factor within the range of the experiment, 17 experiments were determined from three different levels using a statistical software with BBD, 3*3. Then, RSM was performed during extraction. The experiments used X_1_, which represents the ethanol concentration; X_2_, which represents the extraction time; finally, a variable called the X_3_ serves as the solid–liquid ratio. Our dependent or output value is the saponins extracted (Y_O_). The interaction effect of independent variables and response values is usually expressed using the following second-order polynomial Equation 2:

$$Y_{0}=\beta_{0}+\sum_{i=1}^{3}\beta_{i}X_{i}+\sum_{i=1}^{3}\beta_{\mathrm{ii}}X_{i}^{2}+\sum_{i<j}^{3}\beta_{\mathrm{ij}}X_{i}X_{j}+\varepsilon$$ (2)

Results were obtained with Design Expert 8.0.6. A model was used to assess each individual independent variable along with all potential interactions between them at the time of extraction. Equation Y_0_ represents the predicted saponin yield, and *ε* indicates random experimental error. β_0_, β_i_, β_ii_, and β_ij_ denote the intercept, linear, quadratic, and interaction terms, respectively, and X_i_ and X_j_ refer to certain levels (coded or actual) of the independent factor.

### 2.5 UPLC/Q-TOF-MS analysis

The total saponins obtained from hazel mushrooms via UAE were first purified using macroporous resin and subsequently lyophilized. For sample preparation, 0.5 g powder was removed and placed in 10 mL methanol. Then, the reaction mixture was mixed via sonication and passed through a 0.22 μm syringe filter to obtain a clear solution for analysis.

#### 2.5.1 Chromatographic and MS conditions

UPLC/Q-TOF-MS analysis was conducted using an ACQUITY system equipped with an electrospray ionization (ESI) source. Chromatographic separation was performed on an agilent extended C18 column (2.1 × 50 mm^2^, 1.7 μm) protected by a positive-charge mode guard column. The column temperature was held constant at 35 °C, and the injection volume was set to 20 μL. The mobile phase consisted of 5% aqueous acetic acid (A) and acetonitrile (B). MS was performed via an ESI source in positive and negative ion modes with a scan range of m/z 50–1,200. The source conditions were set as follows: desolvation temperature, 150 °C; gas flow, 15 L/min; source (prot.) temperature, 350 °C.

#### 2.5.2 LC–MS analysis method

A total of 20 μL prepared sample was injected into the LC system, and the total ion chromatogram (TIC) and MS data for positive and negative ionizations were recorded.

### 2.6 Cytotoxicity

A549 and MG63 cells were cultured in Dulbecco’s Modified Eagle Medium (10% FBS and 1% penicillin–streptomycin) at 37 °C in 5% CO_2_ incubator. We tested the toxicity of hazel mushroom saponins toward cells by performing MTT test thrice. The cells were plated in 96-well plates at 1 × 10^5^ cells/mL (110 μL/well) and allowed to attach for 24 h. The medium was then changed to fresh DMEM supplemented with saponin samples for 24 h treatment. We then discarded all the medium and exchanged it for 100 μL completely new DMEM and 10 μL MTT solution (5 g/mL). The mixture was incubated for 3 h, the supernatant was removed, and 150 μL dimethyl sulfoxide (DMSO) was dispensed into each well to solubilize formazan crystals. Finally, the absorbance at 490 nm was measured via a microplate reader, and DMSO was used as a negative control.

### 2.7 Apoptosis

A549 and MG63 cell suspensions: 1.25 × 10^5^/mL (2 mL/well). The samples were placed on 6-well plates. The mixture was followed 24 h of incubation, after which the medium was refreshed, and various concentrations (in μL) of hazel mushroom saponins were added to each group (i.e., 25, 50, 100, and 200), with DMSO used as a control. We analyzed the cells for their ability to undergo apoptosis and cell cycle using Annexin V-FITC kits (eBioscience) (19, 20), following the manufacturer’s protocol. The collected cells rinsed twice with ice-cold phosphate-buffered saline (PBS) and then resuspended in 100 μL binding buffer. The samples were incubated with Annexin V-fluorescein isothiocyanate (FITC) and propidium iodide (PI), stained for 15 min at room temperature, and analyzed via flow cytometry immediately.

## 3 Results and discussion

### 3.1 Single-factor experimental analysis

**Figure 1:**
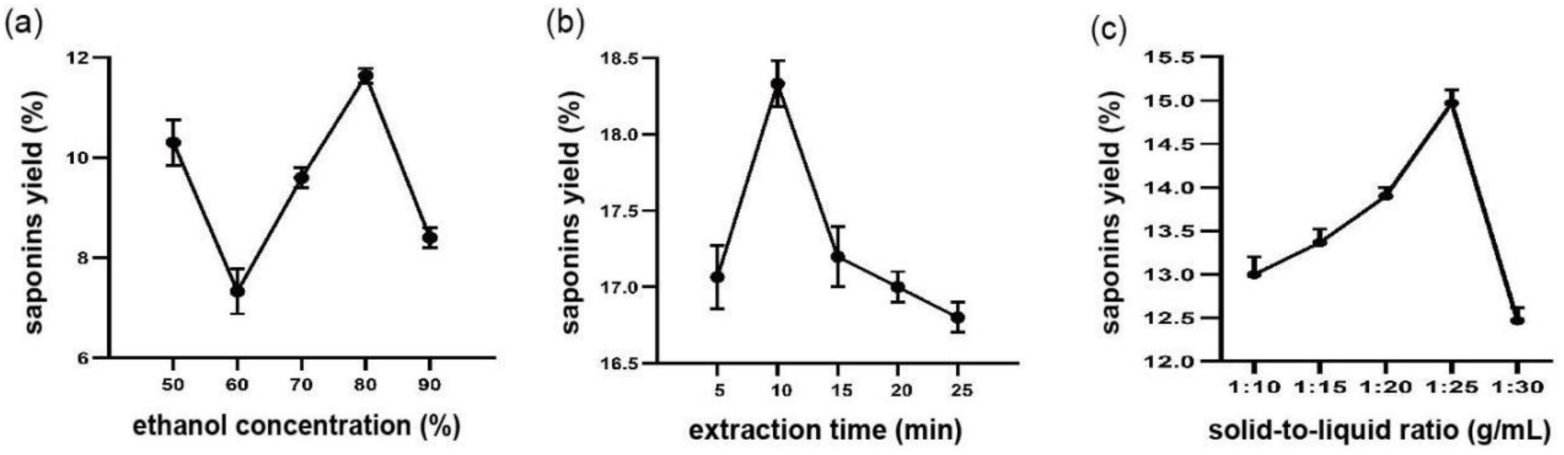
Effects of different **(a)** Ethanol concentration, **(b)** Extraction time and **(c)** Solid-liquid ratio on extraction yield of hazel mushroom saponins. Data are shown as the mean±S.D. from three independent experiments.

When the ethanol concentration was increased from 50 to 80%, the amount of hazel mushroom saponin extract increased. It stopped increasing at 11.63% (Figure 1a) and then declined (Figure 1a). Afterward, the level was lower because the presence of more ethanol favored the dissolution of saponin. However, the sample proteins were denatured or became solid. In addition, we were physically obstructed from accessing the inside of those mushrooms, which halted our progress and resulted in the lower value observed.

The effect of extraction time on the yield of hazel mushroom saponins had a parabolic shape, with a peak observed at 18.33% after 10 min (Figure 1b). During the first 10 min of the experiment, the increase in the amount was due to the increased number of saponins continuously penetrating the membrane and being distributed within the solvent. However, when the ultrasonication time was extended, the extraction efficiency was still poor, possibly because this long-term process caused the breakdowns of heat-sensitive or unstable components, which also resulted in their degradation.

Hazel mushroom saponins were extracted by volume with a bell-shaped profile, and maximum yields (14.97%) were observed at an optimized solid-to-liquid volume ratio of 1:25 g/mL (Figure 1c). Raising the liquid-to-solid ratio improves the contact between the solvent and the raw material, creating a steeper concentration gradient that effectively speeds up dissolution. However, this benefit has its limits; beyond a certain point, the extraction yield often begins to decline. This drop is usually attributed to excessive swelling of the material or the co-extraction of unwanted impurities, both of which can interfere with the diffusion of the target compounds.

### 3.2 RSM (BBD method) optimization

On the basis of the parameters obtained from the single-factor preliminary experiment, this paper used RSM for further optimization of the extraction process. In particular, we used a three-factor, three-level CCD (Table 1). Unlike previous methods, which optimize one thing at a time, these methods consider how factors, such as alcohol content, extraction duration, and the liquid-to-solid ratio, interact very precisely, which hastened the experiments and lessened the time and resources needed.

**Table 1:** Analytical factors and RSM levels.

Levels	Factors		
	Ethanol concentration (%)	Extraction time (min)	Ratio of liquid to solid (mL/g)
−1	70	5	20
0	80	10	25
1	90	15	30

Table 2 shows the results of 17 experimental runs, with saponin yields ranging from 15.36 to 23.56%. To understand how these variables affected the yield, we modeled the data using a second-order polynomial (Equation 3) and determined the regression coefficients to obtain the following relationship:

$$\begin{array}{l} Y=23.04-0.8213X_{1}-0.1763X_{2}-0.9925X_{3}\\ +0.4650X_{1}X_{2}-0.0175X_{1}X_{3}+0.3025X_{2}X_{3}\\ -2.48X_{1}^{2}-3.37X_{2}^{2}-2.91X_{3}^{2} \end{array}$$ (3)

where Y represents the response variable, that is, the extraction yield of saponins from hazel mushrooms, and X_1_, X_2_, and X_3_ indicate the ethanol concentration, extraction time, and liquid-to-solid ratio, respectively. ANOVA was performed, and all the F test results plus the *p* value at Table 3 were used.

**Table 2:** Program and experimental results of RSM.

No.	Ethanol concentration (%)	Extraction time (min)	Ratio of liquid to solid (mL/g)	Saponin yield (%)
1	80	5	30	15.36
2	80	10	25	22.05
3	70	5	25	19.52
4	90	10	20	18.43
5	90	10	30	16.06
6	70	15	25	17.33
7	80	15	20	17.55
8	70	10	30	16.90
9	80	15	30	16.52
10	90	5	25	16.11
11	80	10	25	23.06
12	80	5	20	17.60
13	90	15	25	15.78
14	80	10	25	23.11
15	80	10	25	23.44
16	80	10	25	23.56
17	70	10	20	19.20

**Table 3:** ANOVA of the second-order polynomial model for saponins yield.^a^

Source	Sum of squares	*F*-value	*p*-value
Model	137.02	22.62	0.0002
X_1_	5.40	8.02	0.0253
X_2_	0.2485	0.3693	0.5626
X_3_	7.88	11.71	0.0111
X_1_X_2_	0.8649	1.29	0.2943
X_1_X_3_	0.0012	0.0018	0.9672
X_2_X_3_	0.3660	0.5439	0.4848
$X_{1}^{2}$	25.99	38.62	0.0004
$X_{2}^{2}$	47.95	71.25	<0.0001
$X_{3}^{2}$	35.70	53.06	0.0002
Residual	4.71		
Lack of Fit	3.29	3.10	0.1513
Pure error	1.42		
Cor Total	141.73		
R-Squared	0.9668	Pred R-Squared	0.6124
Adj R-Squared	0.9240	Adeq Precision	11.7699

a Results were obtained with Design Expert 8.0.6.

In Table 3, all the figures indicate very good regression reliability, with a high degree of agreement, as shown by the *R*^2^ (0.9668) and adj-*R*^2^ (0.9240) values. Adequate precision (AP) was achieved at 11.7699, which substantially outperforms the benchmark of 4. This finding lends support to our precise predictive ability during the prediction process. Significance. The results reveal a strong correlation between 2 s-degree variables, namely, the yield of hazel mushroom saponins (X_1_^2^) and (X_2_^2^), with *p* values less than 0.05. Further analysis was performed by creating 3D response surfaces and contour map plots to visualize how these variables interacted with each other and contributed to our yield values.

**Figure 2:**
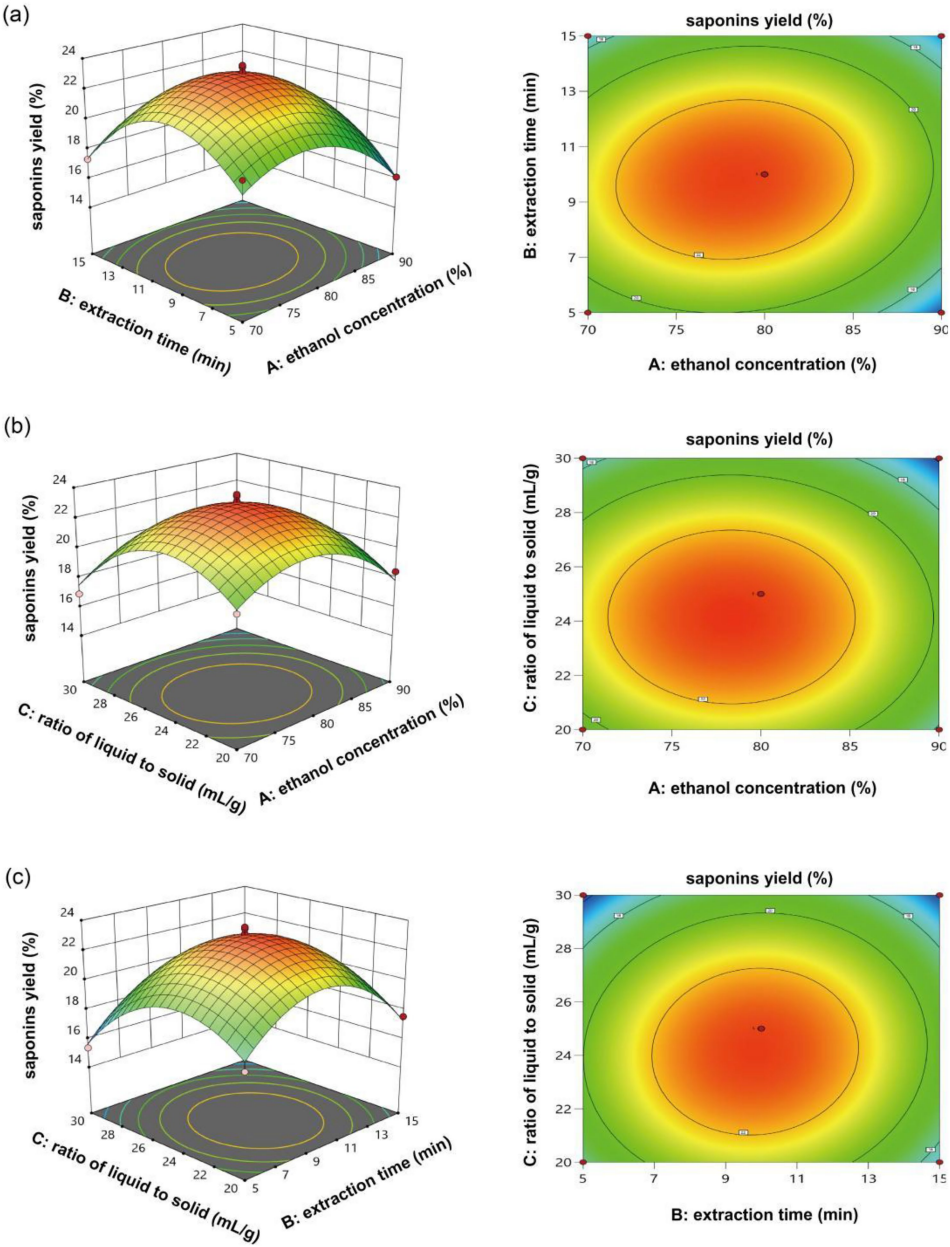
**(a–c)** Response surface and corresponding contour plots illustrating the interaction effects between various influencing factors.

Figure 2a shows the 3D response surface plot (left) and 2D contour plot (right) revealing the effects of ethanol concentration and extraction time on the extraction yield of saponins from hazel mushrooms. With the increase in time and ethanol, the yield increased and then decreased again. The response surface was noticeably arched with elliptical contour lines, which suggests a strong interaction between extraction time and ethanol concentration. The high curvature of the response surface indicates that the extraction yield was very sensitive to small changes in these variables. Figures 2b,c also show clear interactions between ethanol concentration and solid–liquid ratio and between extraction time and solid–liquid ratio. In addition, the extraction yield initially increased before decreasing in both cases. The best conditions for extracting saponins from hazel mushrooms were an ethanol concentration 73.28%, a solid–liquid ratio 1:24.88 g/mL, and an extraction time 12.5 min, resulting in a yield of 21.4%. Therefore, we can increase the amount of saponin obtained from hazel mushrooms by modifying important variables, such as the duration, amount of water used compared with mushrooms, and amount of alcohol added to the mixture.

### 3.3 UPLC/Q-TOF-MS identification of hazel mushroom saponins

**Figure 3:**
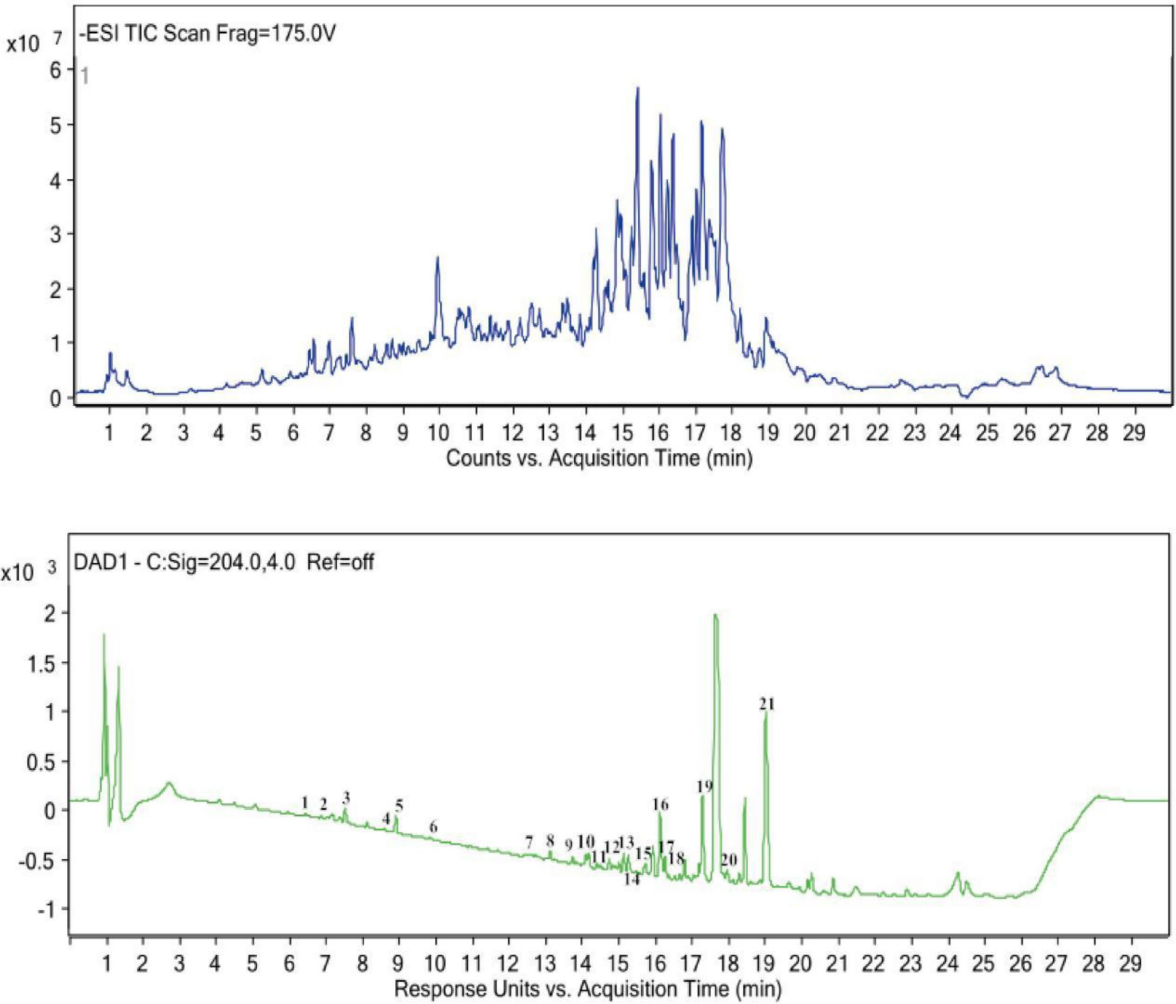
Base peak chromatogram (BPC) of saponins from hazel mushrooms acquired via UHPLC-ESI-Q-TOF-MS analysis.

In negative ionization mode, the ionization efficiency of saponins derived from hazel mushrooms was significantly greater than that in positive ionization mode. Thus, negative ionization mode was prioritized for qualitative analysis. By synthesizing the data acquired from UPLC–Q–TOF–MS, including the TIC (21, 22), relative retention times, mass–charge ratios (m/z), and secondary fragment ions, and performing a comparative analysis with established literature, the chemical constituents of these saponins were systematically characterized. Figure 3 shows the negative-ion-mode TIC of the saponin extract obtained via UAE. According to the results of UPLC–Q-TOF–MS analysis, 21 chemical components, including 14 sesquiterpenes, 1 adenosine, 3 sterols, 2 diterpenoids/triterpenoids, and 1 purine (Table 4), were determined in the UAE product.

**Table 4:** Analysis of the constituent of hazel mushroom saponins.

No	Rt (min)	Detected m/z[M-H]^**−**^	Calculated m/z[M-H]^**−**^	Mass error (ppm)	Formula	MS/MS (MS^E^)m/z	Compounds identification
1	6.409	395.1853	397.1859	−0.0017	C_24_H_28_O_5_	396.1917	Armillaricin
2	6.692	295.1308	296.1362	−0.0007	C_12_H_17_N_5_O_4_	295.1307	N,N-dimethyladenosine
3	7.606	420.2159	422.2084	−0.0032	C_23_H_32_O_7_	420.2132	Armillarizin
4	8.687	447.165	449.1545	−0.0013	C_23_H_28_O_9_	448.1713	Melledonal E
5	8.836	433.1904	435.2020	−0.0077	C_23_H_30_O_8_	434.1977	Melledonol
6	9.568	313.1673	316.2072	−0.0013	C_20_H_26_O_3_	314.1913	7-oxodehydroabietic acid
7	12.378	415.2033	418.2191	0.0037	C_24_H_32_O_6_	416.2163	Armilly everninate
8	12.893	383.1883	385.2012	−0.0062	C_23_H_28_O_5_	384.1962	Armillarivin
9	13.442	462.1826	463.1910	−0.0016	C_25_H_32_ClO_6_	463.1916	Arnamial
10	14.073	395.3298	398.3395	0.0031	C_28_H_44_O	396.3369	Ergosterol
11	14.489	451.1447	454.1486	0.0072	C_23_H_29_ClO_7_	452.1528	Melleolide M
12	14.605	413.1983	415.2094	−0.0062	C_24_H_30_O_6_	414.2062	Armillarin
13	14.938	401.1909	404.2007	0.0019	C_23_H_30_O_6_	402.1981	4-dehydrodihydromelleolide
14	15.221	427.3198	430.3222	0.0040	C_28_H_44_O_3_	428.3260	Ergosterol peroxide
15	15.387	431.1718	434.1873	0.0030	C_23_H_28_O_8_	432.177	Melledonal
16	16.268	445.1921	447.1947	−0.0091	C_24_H_30_O_8_	446.1991	Armillarisin
17	16.386	447.197	449.2065	0.0079	C_24_H_32_O_8_	448.2021	Melleolide C
18	16.584	301.2188	303.2295	−0.0062	C_20_H_30_O_2_	302.2262	Pimaric acid
19	17.216	413.3292	416.3747	0.0030	C_28_H_46_O_2_	414.347	5,6-epoxy-3-hydroxy-ergosterol
20	17.931	429.1967	431.1994	−0.0035	C_24_H_30_O_7_	430.2035	Armillarigin
21	18.895	399.1858	401.1893	−0.0028	C_23_H_28_O_6_	400.1928	Melleolide

### 3.4 Cytotoxicity results

**Figure 4:**
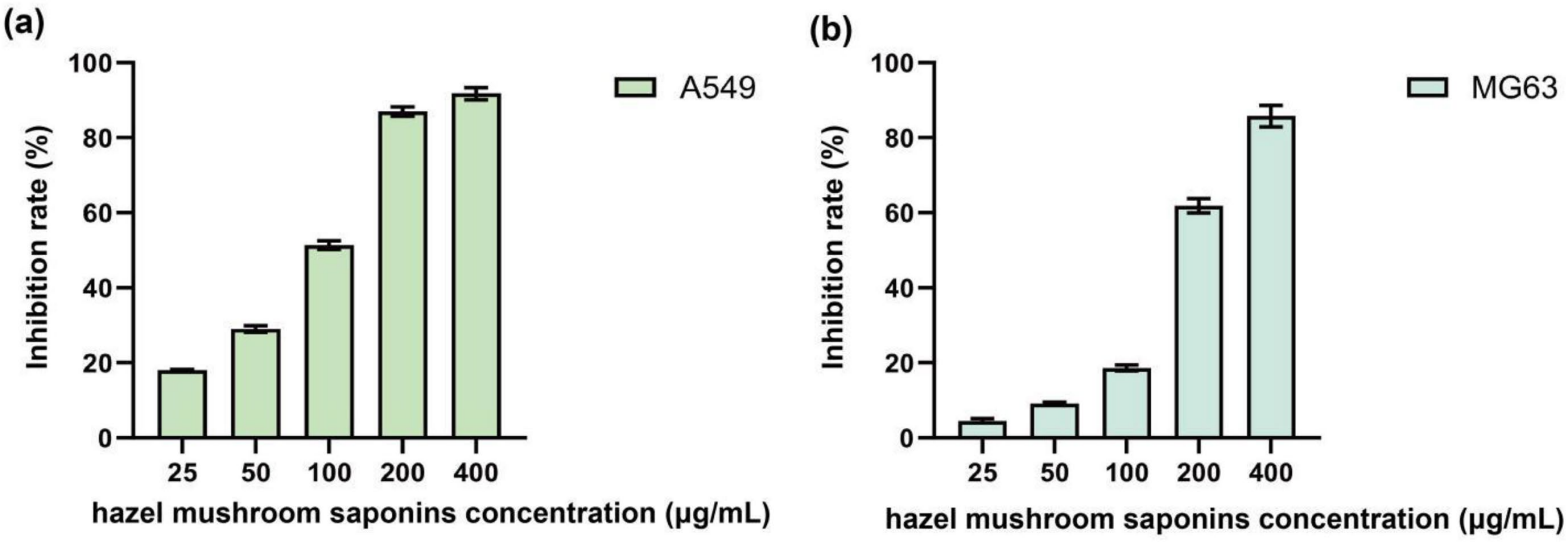
Inhibitory effect of saponins extracted from hazel mushroom on tumor cell A549 **(a)** and lung cancer MG63 **(b)**.

Lung cancer is currently one of the malignant tumors with the highest incidence and mortality rates worldwide (23, 24). At present, clinical treatment for lung cancer relies primarily on chemotherapy and radiotherapy and emerging targeted therapy and immunotherapy. However, the therapeutic outcomes remain suboptimal. Osteosarcoma is the most common type of primary malignant bone tumor in adolescents; it is very invasive and tends to metastasize to the lungs at an early stage (25, 26). First-line chemotherapeutic agents, such as methotrexate, adriamycin, and cisplatin, if used in the long term, lead to the development of multidrug resistance and treatment failure. In addition, natural products, another enormous class of anticancer drugs, such as vincristine and camptothecin, used clinically are derived from plants. Saponins, which are active substances, are common in several costly traditional Chinese medicine herbs, such as *Panax ginseng*, Paris polyphylla, Panax notoginseng, and bupleurum. Scholars have shifted their focus on them because of their complex chemical structure and powerful biological activity. According to research, saponins have antitumor properties that target various locations and process, but they cause no damage to our body. Effects of different concentrations of hazel mushroom saponins on the inhibition rate of A549 cells (Figure 4a). Hazel mushroom saponins restricted the proliferation of A549 cells; when the concentration was increased from 25 μg/mL to 400 μg/mL, the inhibition rate of A549 cells increased rapidly from 18.08 to 91.75%. Effects of different concentrations of hazel mushroom saponins on the inhibition rate of MG63 cells (Figure 4b). As the concentration of hazel mushroom saponins increased, the inhibition rate against MG63 cells increased progressively, reaching 62.88% at a concentration of 200 μg/mL.

### 3.5 Effects of hazel mushroom saponins on tumor cell apoptosis

**Figure 5:**
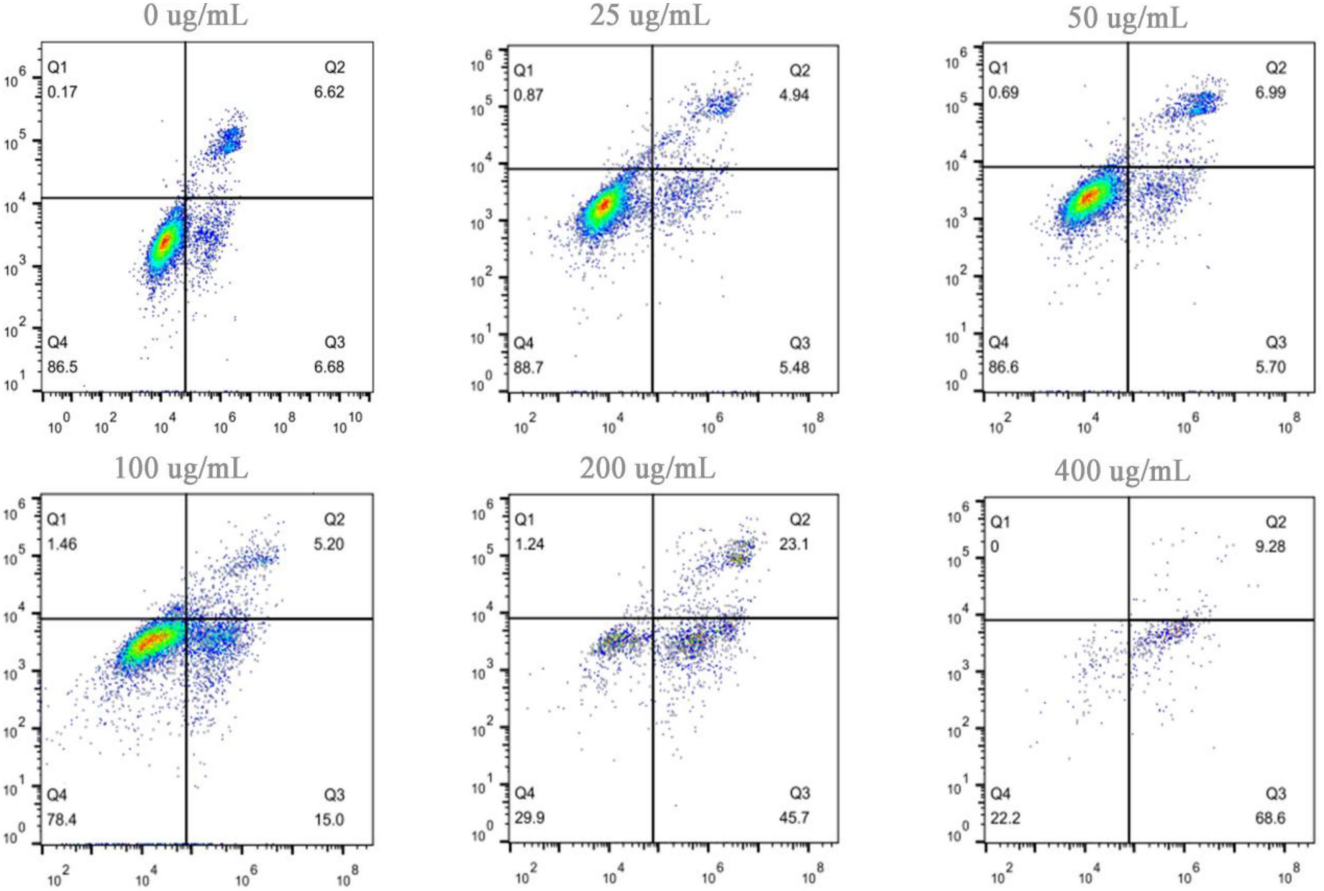
Apoptosis induction in A549 cells by saponins from hazel mushrooms.

**Figure 6:**
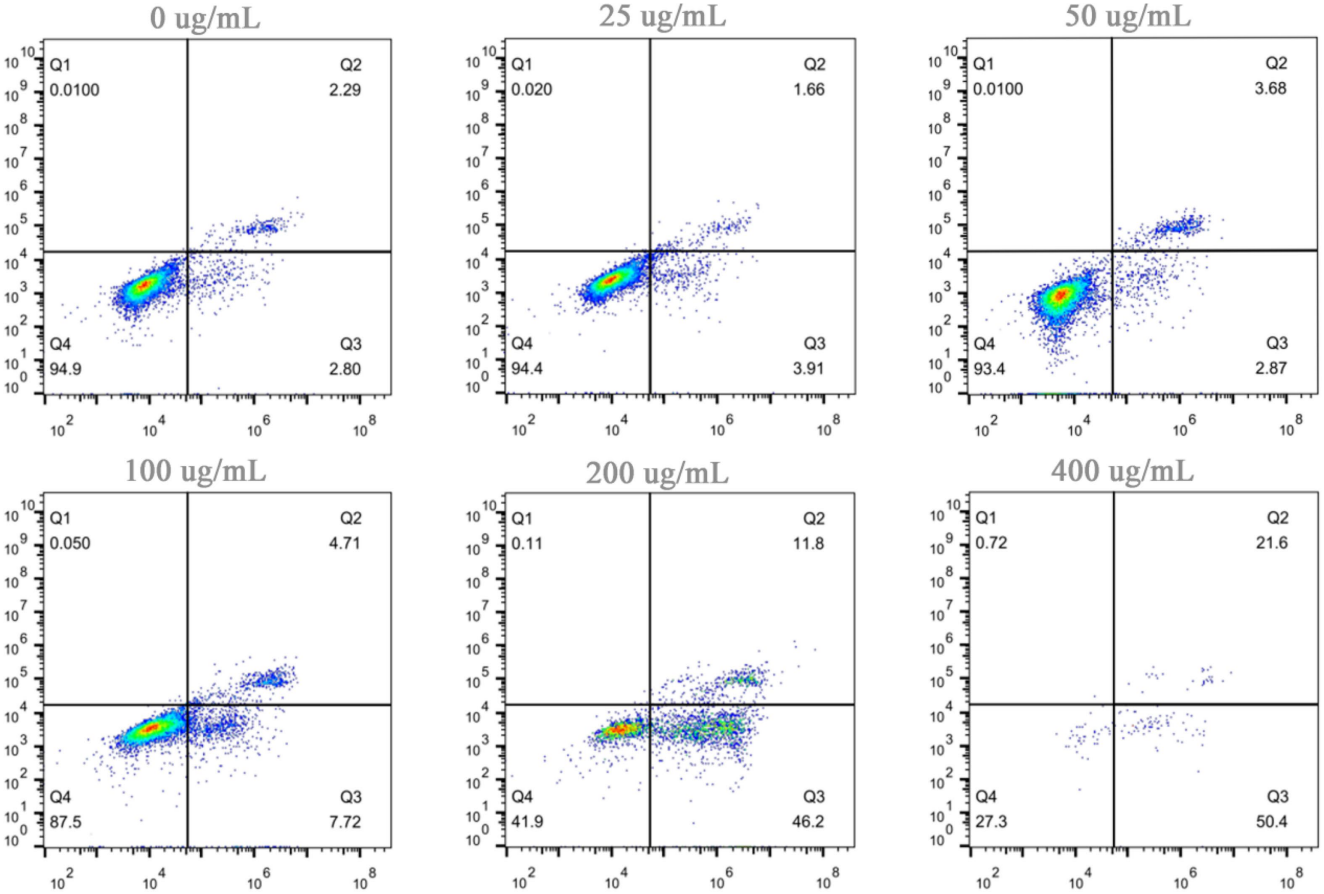
Apoptosis induction in MG63 cells by saponins from hazel mushrooms.

Apoptosis is a genetically controlled process of programmed cell death, and it plays a pivotal role in maintaining internal homeostasis. Tumorigenesis and progression are largely attributed to the evasion of apoptosis, which grants abnormal cells the capacity for unlimited proliferation. Consequently, reactivating the apoptotic program in tumor cells via exogenous or endogenous pathways is widely recognized as one of the most effective strategies for developing anticancer drugs. The proapoptotic effects of hazel mushroom saponins on A549 and MG63 cells were determined via Annexin V-FITC/PI staining and flow cytometry. The experimental results in Figure 5 demonstrate that after 24 h, compared with those of the control group, the total apoptosis rates of the A549 cells reached 68.8% (45.7% early apoptosis) and 77.88% (68.6% early apoptosis) at final concentrations of 200 and 400 μg/mL, respectively. Similarly, Figure 6 shows that after 24 h, the total apoptosis rates of MG63 cells reached 58% (46.2% early apoptosis) and 72% (50.4% early apoptosis) at the same concentrations. These findings indicate that hazel mushroom saponins significantly induced apoptosis in tumor cells, with most cells remaining in the early apoptotic stage after 24 h, suggesting a relatively rapid onset of action. The apoptotic process involves intricate signal transduction pathways, including the mitochondrial (endogenous), death receptor (exogenous), and endoplasmic reticulum stress pathways, which provide numerous targets for antitumor research. The mechanisms of action of many natural products are not yet fully understood, and their molecular targets for specific tumor types require further exploration. Elucidating how hazel mushroom saponins regulate apoptotic signaling pathways to exert their antitumor effects remains a scientific question to be resolved. To date, our investigations have been restricted to *in vitro* antitumor activity; further *in vivo* studies are required to substantiate these findings. Notably, hazel mushrooms possesses distinct competitive advantages over conventional medicinal fungi—such as *Ganoderma lucidum* and Ginseng—owing to its favorable safety profile, economic feasibility, and dual-purpose utility as a food and therapeutic agent, positioning it as a highly competitive candidate in the pharmacological market.

## 4 Conclusion

Here, we used ultrasonic waves to extract total saponins from hazel mushrooms and optimize the extraction process with RSM. The optimal extraction conditions were as follows: ethanol, 73.28%; solid–liquid ratio of 1:24.88 g/mL, and a maximum extraction time of 12.5 min, with a yield of 21.4%. The results of in vitro tumor cell inhibition experiments indicate that when the concentration of hazel mushroom saponin was greater than 200 μg/mL, the inhibition rates of A549 and MG63 cells were greater than 50%. Saponins mostly cause tumor cells to undergo early apoptosis. However, further investigation is needed to determine the causes of such a phenomenon. In addition, the main components of the ultrasonically extracted saponins were roughly identified via UPLC–Q–TOF–MS, which provided a basic theory for investigating their pharmacodynamics. Compared with ginseng-type medicinal materials such as ginseng, which take many years to grow, edible fungi have long standing food safety, shorter cultivation period, and greater yields. Currently, our research has not made any real progress. By taking advantage of their safety features, more emphasis should be placed on “functional enhancement” over plain old “extract,” and support should be given to individuals prone tumors through a type of food that they can consume regularly.

## Data Availability

The original contributions presented in the study are included in the article/supplementary material, further inquiries can be directed to the corresponding authors.
